# 

**Published:** 1874-01

**Authors:** 


VOLUME XI. | JANUARY—1874. | NUMBER 10.

### ATLANTA

### Medical and Surgical Journal.

MONTHLY: THREE DOLLARS PER ANNUM, IN ADVANCE.

ROBERT BATTEY, M.D.,

EDITOR.

WM. ABRAM LOVE, M.D., V. H. TALIAFERRO, M.D.,
ASSOCIATE EDITORS.

*PAX ET SCIENTIA, SED VERITAS SINE TIMORE.*

ATLANTA, GEORGIA:

*W. R. BARROW, BOOK AND GENERAL JOB PRINTER.*

1874.

